# Acute vascular complications of femoral veno-arterial ECMO: a single-centre retrospective study

**DOI:** 10.1186/s43044-021-00143-y

**Published:** 2021-02-19

**Authors:** Mohamed Laimoud, Elias Saad, Samer Koussayer

**Affiliations:** 1 Adult Cardiac Surgical Intensive Care Department, King Faisal Specialist Hospital & Research Center, Riyadh, Saudi Arabia; 2 Critical Care Medicine Department, Cairo University, Cairo, Egypt; 3 Vascular and Endovascular Surgery Department, King Faisal Specialist Hospital & Research Center, Riyadh, Saudi Arabia; 4 College of Medicine, Alfaisal University, Riyadh, Saudi Arabia

**Keywords:** Veno-arterial ECMO, Femoral, Limb ischemia, Percutaneous cannulation, Ultrasound, Cut-down

## Abstract

**Background:**

Femoral arterial cannulation to initiate veno-arterial ECMO may result in ipsilateral limb ischemia due to reduced distal blood flow below the insertion point of the cannula. We retrospectively studied adult patients supported with femoral VA-ECMO for cardiogenic shock between 2015 and 2019 at our tertiary care hospital.

**Results:**

The study included 65 adult patients supported with femoral VA-ECMO for refractory cardiogenic shock. The studied patients had a mean age of 37.9 ± 14.87 years, mostly males (70.8%), a mean BSA of 1.77 ± 0.27 m^2^, and a mean BMI of 26.1 ± 6.7 kg/m^2^. Twenty-one (32.3%) patients developed acute lower limb ischemia. The patients who developed acute limb ischemia had significantly frequent AKI (< 0.001) without significant use of haemodialysis (*p* = 0.07) and longer ICU stay (*p* = 0.028) compared to the patients without limb ischemia. The hospital mortality occurred in 29 (44.6%) patients without significant difference between the patients with and without acute limb ischemia. The occurrence of acute limb ischemia was significantly correlated with failed percutaneous cannulation (*p* = 0.039), while there was no significant statistical correlation between the cut-down technique and occurrence of limb ischemia (*p* = 0.053). The occurrence of femoral cannulation site bleeding was significantly correlated with failed percutaneous cannulation (*p* = 0.001) and cut-down technique (*p* = 0.001).

**Conclusion:**

Acute vascular complications are frequent after femoral VA-ECMO. Failed percutaneous femoral cannulation has been, in this study, identified as the most important risk factor for acute limb ischemia and cannulation site bleeding. A careful approach during femoral cannulation is recommended to prevent occurrence of acute limb ischemia and femoral cannulation site bleeding.

## Background

Emergent veno-arterial extracorporeal membrane oxygenation (VA-ECMO) is increasingly used to provide rapid cardiopulmonary resuscitation in adult patients with refractory cardiogenic shock [1–3]. The emergent nature of the haemodynamics deterioration, as in cardiac arrest or post-cardiotomy shock conditions, makes peripheral ECMO initiation via femoral vessels the preferred approach. Either percutaneous or surgical cut-down approaches are being used for emergent femoral cannulation to initiate VA-ECMO support [4, 5]. Femoral arterial cannulation may result in ipsilateral limb ischemia due to reduced distal blood flow below the insertion point of the cannula [6–8]. Recent studies have demonstrated that the development of acute limb ischemia had a bad impact on patient mortality and quality of life of the survivors after ECMO decannulation [9, 10]. We conducted this retrospective study to report and analyse the vascular complications of peripheral VA-ECMO in the adult patients with cardiogenic shock in our tertiary care hospital.

## Methods

### The study design

The ethics committee board of our institute approved this retrospective study. We included adult patients who were supported with femoral veno-arterial ECMO for cardiogenic shock between 2015 and 2019 at our tertiary care hospital. The enrolled patients were divided according to occurrence of acute lower limb ischemia into 2 groups. The hospital electronic database was used to get the clinical and laboratory variables of studied patients. We assessed the studied patients using SOFA score upon ICU admission and then at the third and fifth days. The Δ1 SOFA was the difference between SOFA scores at third and admission days. The Δ2 SOFA was the difference between SOFA scores at the fifth and admission days [11–13].

### The femoral cannulation technique

The femoral arterial and venous cannulations were done via either percutaneous or cut-down approaches according to the surgeons preferences. Seldinger technique was use to cannulate the femoral vein and a 19–23-Fr cannula was introduced over a guidewire till the inferior vena cava (IVC). The common femoral artery was similarly cannulated with a 15–19-Fr cannula. Insertion of an additional distal 6-Fr cannula was also performed to preserve lower limb perfusion in most of enrolled patients. The selection of type and size of the arterial cannula was selected according to the patient’s body surface area (BSA) and the required ECMO flow which is equivalent to a cardiac index (CI) of 2.2–2.5 L/m^2^/min [14].

### The ECMO management and decannulation protocol

After VA-ECMO initiation, the blood flow was adjusted based on clinical evaluation including mean arterial blood pressure, urine output, clearance of blood hyperlactatemia, and mixed venous oxygen saturation. Titration of oxygen and sweep flows was  gradually done to achieve acceptable blood gases. Minimization of the doses of vasopressors intravenous infusions was done to decrease risk of limb ischemia. Anticoagulation was done via intravenous heparin infusion which was adjusted according to heparin assay (target 0.3–0.7 units/ml), antithrombin (AT) III (goal 80–120%), and clinical tolerance. Platelets transfusion to keep count more than 50 (10^9^/L), packed red blood corpuscles transfusion to maintain the haemoglobin level above 80 (gm/L) were done. Monitoring of the lower limbs perfusion was routinely done by clinical and Doppler ultrasound assessments. The clinical assessment includes skin temperature and appearance to detect any coldness, pallor or mottling compared to contralateral limb, and cannulation site bleeding or haematoma. Doppler ultrasound assessment was used to detect the peak systolic velocity of dorsalis pedis and posterior tibial arteries. Near-infrared spectroscopy (NIRS) monitoring is routinely applied to all patients with femoral VA-ECMO to provide continuous monitoring cerebral oxygenation via bilateral frontal probes. Also, NIRS was used in patients with suspected limb impaired perfusion via limb probes. NIRS was proved to be effective in continuous monitoring of limb regional oxygen saturation even with absence of pulsatile flow [15, 16]. For any suspicion of limb ischemia, complete involvement of vascular surgery team was done. Removal of femoral cannulae was done after exposing the femoral vessels. The femoral vessels were primarily repaired. In case of limb ischemia, vascular surgical interventions were done including thrombectomy and angioplasty. Fasciotomy was done in case of acute leg compartmental syndrome.

### The statistical analysis

Data was coded and entered using the Statistical Package for the Social Sciences (SPSS) version 26 (IBM Corp., Armonk, NY, USA). Data was presented using mean (± standard deviation) or median (interquartile range) in quantitative data and using frequency (and percentage) for categorical data. Comparisons between quantitative variables were done using the Mann-Whitney test. For comparing categorical data, Chi square (*χ*^2^) test was performed. Graphs were used to illustrate some information. *p* values less than 0.05 were considered statistically significant.

## Results

### Baseline clinical variables of studied patients

The study included 65 adult patients supported with femoral VA-ECMO for refractory cardiogenic shock. The studied patients had a mean age of 37.9 ± 14.87 years, mostly males (70.8%), a mean BMI of 26.1 ± 6.7 kg/m^2^ and a mean BSA of 1.77 ± 0.27 m^2^. Twenty-one (32.3%) patients developed acute lower limb ischemia. There were no statistically significant differences between the patients with and without limb ischemia regarding demographic data, clinical variables, size of ECMO cannulae, prophylactic distal perfusion cannula (DPC) insertion ultrasound-guided approach, and cut-down approaches. Failed percutaneous cannulae insertion was statistically significant in the patients who developed acute limb ischemia (Table 1).

**Table 1:** Baseline clinical variables of the VA-ECMO-supported patients

Studied criteria	All patients (*n* = 65)	Limb ischemia group (*n* = 21)	No limb ischemia group (*n* = 44)	*P* value
Age (years)	37.9 ± 14.87	34.4 ± 12.3	39.6 ± 15.8	0.19
Sex				
Males	46 (70.8)	18 (85.7)	28 (63.6)	0.06
Females	19 (29.2)	3 (14.3)	16 (36.4)	
Weight (kg)	70.88 ± 20.9	70.6 ± 23.4	71 ± 19.9	0.61
Height (cm)	164.68 ± 8.1	164.57 ± 6.3	164.7 ± 8.9	0.83
BMI (kg/m^2^)	26.08 ± 6.7	26.04 ± 7.7	26.11 ± 6.4	0.43
BSA (m^2^)	1.77 ± 0.27	1.76 ± 0.29	1.78 ± 0.27	0.54
Diabetes mellitus	13 (20)	5 (23.8)	8 (18.2)	0.74
Hypertension	18 (27.7)	5 (23.8)	13 (29.5)	0.62
Pre-ECMO AF	17 (26.2)	4 (19)	13 (29.5)	0.36
Anticoagulants intake	18 (27.7)	5 (23.8)	13 (29.5)	0.62
Aspirin intake	3 (4.6)	1 (4.8)	2 (4.5)	1
Left ventricle EF	26.75 ± 13.4	26.14 ± 12.1	27.1 ± 14.2	0.81
CKD	10 (15.4)	5 (23.8)	5 (11.4)	0.27
Previous CVS	2 (3.1)	1 (4.8)	1 (2.3)	0.54
Cardiothoracic surgery	17 (26.2)	6 (28.6)	11 (25)	0.75
IABP	13 (20)	4 (19)	9 (20.5)	1
Percutaneous cannula insertion				
Successful	36 (55.4)	8 (38.1)	28 (63.6)	0.039
Failed	19 (29.2)	10 (47.6)	9 (20.5)	
Not tried	10 (15.4)	3 (14.3)	7 (15.9)	
US-guided cannula insertion	32 (49.2)	7 (33.3)	25 (56.8)	0.07
Femoral cut-down	29 (44.6)	13 (61.9)	16 (36.4)	0.053
Insertion at operation room	30 (46.2)	13 (61.9)	17 (38.6)	0.078
Arterial cannula size (Fr)	17.85 ± 1.4	18.05 ± 1.36	17.75 ± 1.43	0.77
Venous cannula size (Fr)	21.72 ± 1.3	21.6 ± 1.59	21.7 ± 1.22	0.82
Distal perfusion cannula	52 (80)	15 (71.4)	37 (84.1)	0.32
Cannulation site bleeding	16 (24.6)	6 (28.6)	10 (22.7)	0.6
Cannulation bleeding required exploration	4 (6.3)	1 (4.8)	3 (6.8)	1

Data are presented as number (%) or mean (± SD)

### The vascular complications of studied patients

**Fig. 1:**
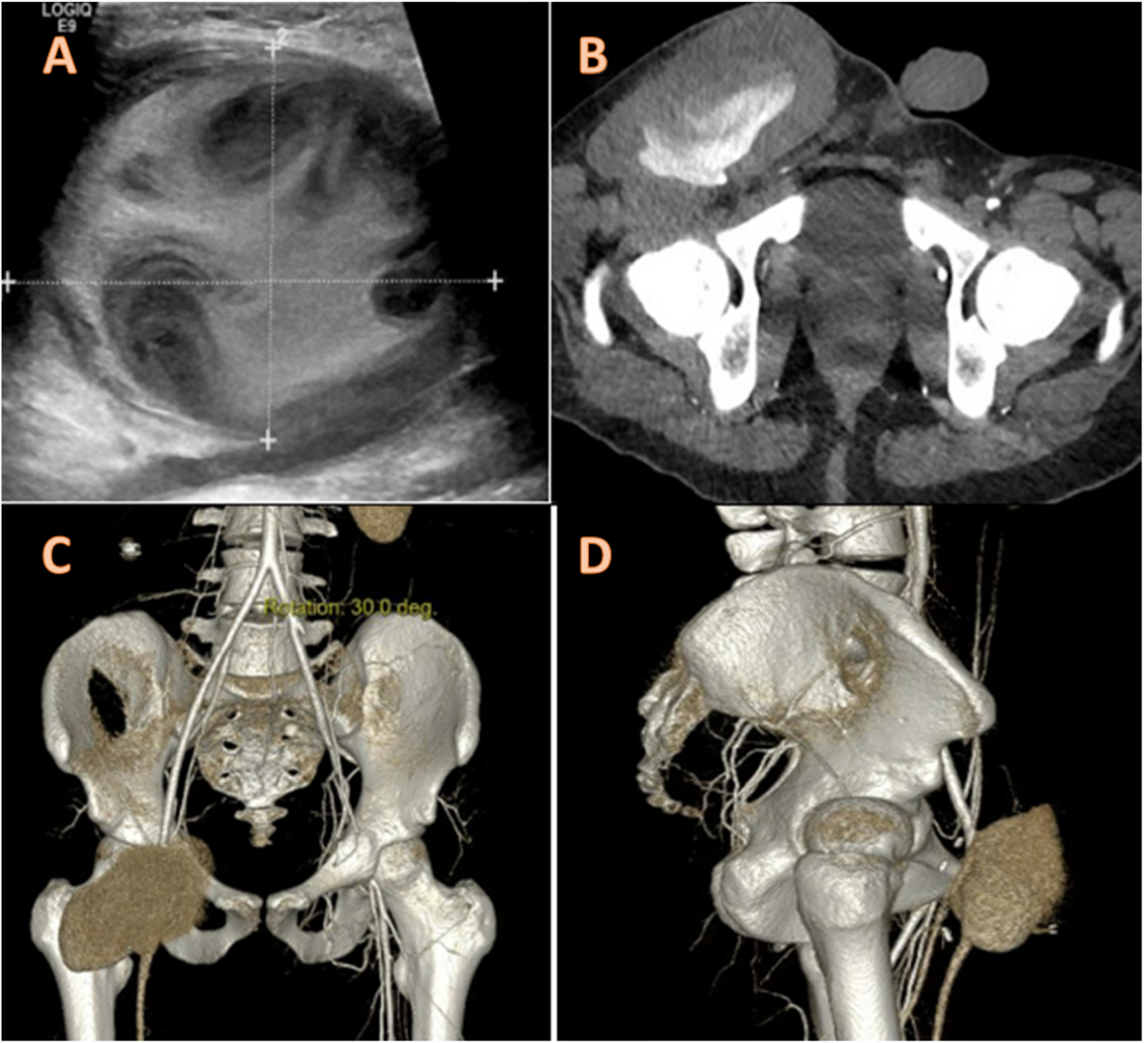
A 21-year-old male patient developed acute right groin swelling after right femoral ECMO decannulation. Groin US revealed a large swelling (7 × 5 cm) with heterogenous echogenicity in the right groin area arising from right femoral artery with the neck measuring 0.6 cm (**a**). CT angiography revealed the large pseudo-aneurysm (12 × 6 cm) from the right common femoral artery with active contrast extravasation (**b**). Multiplanar reconstruction and advanced 3-D postprocessing were performed and revealed the large pseudo-aneurysm with good distal arterial flow (**c**, **d**). Vascular surgery was done with evacuation of the pseudo-aneurysm and repair of the femoral artery

Femoral thrombectomy and angioplasty were done in 20 (30.8%) patients. Four (6.2%) patients developed limb compartmental syndrome and fasciotomy was done. Amputation of toes was done in one patient. The vascular complications included femoral arteriovenous fistula after ECMO decannulation and required vascular surgical repair in one patient. Femoral large pseudo-aneurysm occurred in one patient and required surgical intervention after ECMO decannulation. Three (4.6%) patients developed chronic limb ischemia manifestations during follow-up. Femoral wound infection occurred in 2 (3.1%) patients and complicated the healing process and required sartolius muscle flapping (Table 2, Fig. 1).

**Table 2:** The vascular complications of the studied patients

The vascular complications	Number (%)
Acute limb ischemia	21 (32.3)
Thrombectomy	20 (30.8)
Angioplasty	20 (30.8)
Fasciotomy	4 (6.2)
Toes amputation	1 (1.5)
Cannulation site bleeding	16 (24.6)
Cannulation bleeding required exploration	4 (6.3)
Femoral arteriovenous fistula	1 (1.5)
Femoral pseudo-aneurysm	1 (1.5)
Ipsilateral chronic limb ischemia	3 (4.6)
Groin wound grafting	2 (3.1)

### Laboratory criteria of studied VA-ECMO-treated patients

The patients who develop limb ischemia had significantly higher INR (1.9 (1.4–3.2) vs 1.4 (1.1–3.6), *p* = 0.004) and AT III level (69 (27–88) vs 55.5 (42–73), *p* = 0.002) at time of ECMO initiation compared to those who did not develop ischemia. The serum creatinine was 115 (48–298) vs 81 (9.5–320) (*p* = 0.01), while the serum albumin was 37.4 (22.2–44.1) vs 31.1 (18.3–44.1) (*p* = 0.04) in the limb ischemia and no limb ischemia groups, respectively. There were no significant differences between both groups regarding other laboratory variables including degree of lactic acidosis and lactate clearance after 24 h of ECMO support (Table 3).

**Table 3:** Laboratory criteria of the studied VA-ECMO-treated patients

Studied criteria	All patients	Limb ischemia group	No limb ischemia group	*P* value
Haemoglobin (g/L)	111 (88–166)	105 (88–153)	111 (88–166)	0.9
Platelet count (10^9^/L)	181 (38–447)	160 (112–329)	215 (38–447)	0.32
INR	1.6 (1.1–3.6)	1.9 (1.4–3.2)	1.4 (1.1–3.6)	0.004
aPTT (seconds)	42.2 (31.3–150)	48.2 (33.9–150)	41.3 (31.3–74.3)	0.06
PTT ratio	1.2 (0.9–4.4)	1.4 (1–4.4)	1.2 (0.9–2.2)	0.13
Fibrinogen (g/L)	3.16 (0.76–6.87)	2.38 (0.76–6.87)	3.39 (1.37–6.06)	0.28
Antithrombin III	58 (27–88)	69 (27–88)	55.5 (42–73)	0.002
Serum creatinine (μmol/L)	91 (9.5–320)	115 (48–298)	81 (9.5–320)	0.01
Serum albumin (g/L)	32.2 (18.3–44.1)	37.4 (22.2–44.1)	31.1 (18.3–44.1)	0.04
Serum bilirubin (μmol/L)	28.6 (5.8–389)	58.7 (5.8–79.7)	53.5 (7.5–389)	0.71
PH	7.17 (6.9 –7.34)	7.12 (6.9 – 7.34)	7.20 (6.9 – 7.34)	0.69
Base excess	− 9.3 (− 18.6 to – 4.8)	− 11.2 (− 18.6 to − 5.3)	− 8.25 (− 16.4 to – 4.8)	0.25
Blood lactate (mmol/L)	4.8 (2.4–12.4)	4.8 (3.6–12.4)	5.2 (2.4–10.7)	0.91
Peak blood lactate	13.2 (6.7–20)	13.2 (7.4–20)	11.2 (6.7–20)	0.08
Blood lactate at 24 h	3.1 (1.1–20)	3.1 (1.1–14.3)	2.9 (1.1–20)	0.27

Data are presented as median (IQR)

### Hospital course of studied VA-ECMO-treated patients

The hospital mortality occurred in 29 (44.6%) patients without significant difference between both groups. The patients who developed acute limb ischemia had significantly frequent acute kidney injury (AKI) (< 0.001) without significant use of haemodialysis (*p* = 0.07) and longer ICU stay (*p* = 0.028) compared to the patients without limb ischemia. The median initial SOFA score was 10 (8–21) vs 14 (8–21) (*p* = 0.008) in the limb ischemia and no limb ischemia groups, respectively, without significant differences in the follow-up *∆* SOFA scores at the third and fifth days. There were no significant differences between both groups regarding cerebrovascular strokes, gastrointestinal bleeding, occurrence of new AF, ECMO, and ventilator days (Table 4).

**Table 4:** Hospital course of the studied VA-ECMO-treated patients

Studied criteria	All patients	Limb ischemia group	No limb ischemia group	*P* value
Acute Kidney injury	39 (60)	21 (100)	18 (40.9)	< 0.001
Renal replacement therapy	27 (41.5)	12 (57.1)	15 (34.1)	0.07
Cerebrovascular stroke	13 (20)	5 (23.8)	8 (18.2)	0.74
Cerebral ischemic stroke	7 (11.9)	3 (17.6)	4 (9.5)	0.39
Intracerebral haemorrhage	6 (10.2)	2 (11.8)	4 (9.5)	0.9
ICU days	19 (3–191)	19 (10–93)	17 (3–191)	0.028
Ventilator days	10 (2–191)	14 (5–81)	9 (2–191)	0.053
Gastrointestinal bleeding	14 (21.5)	4 (19)	10 (22.7)	0.92
Atrial fibrillation	25 (38.5)	9 (42.9)	16 (36.4)	0.61
Intracardiac thrombi	4 (6.2)	1 (4.8)	3 (6.8)	0.9
ECMO circuits thrombi	4 (6.2)	1 (4.8)	3 (6.8)	0.9
ECMO days before ischemia	6 (1–21)	6 (1–21)	-------	----
ECMO days	8 (3–40)	7 (4–40)	8 (3–32)	0.27
Changing to central VA-ECMO	3 (4.6)	2 (9.5)	1 (2.3)	0.29
Changing to LVAD	11 (16.9)	4 (19)	7 (15.9)	0.34
Decannulation and no MCS	51 (78.5)	15 (71.4)	36 (81.8)	0.41
Hospital mortality	29 (44.6)	9 (42.9)	20 (45.5)	0.84
SOFA score day 1	12 (8–21)	10 (8–21)	14 (8–21)	0.008
SOFA score day 3	13 (6–24)	10 (7–22)	14 (6–24)	0.034
∆ 1 SOFA	− 1 (− 5 to 6)	1 (− 4 to 6)	− 2 (− 5 to 6)	0.12
SOFA score day 5	10 (5–24)	9 (6–21)	10 (5–24)	0.45
∆ 2 SOFA	− 2 (− 7 to 9)	0 (− 5 to 8)	− 3 (− 7 to 9)	0.14

Data are presented as number (%) or median (IQR)

**Fig. 2:**
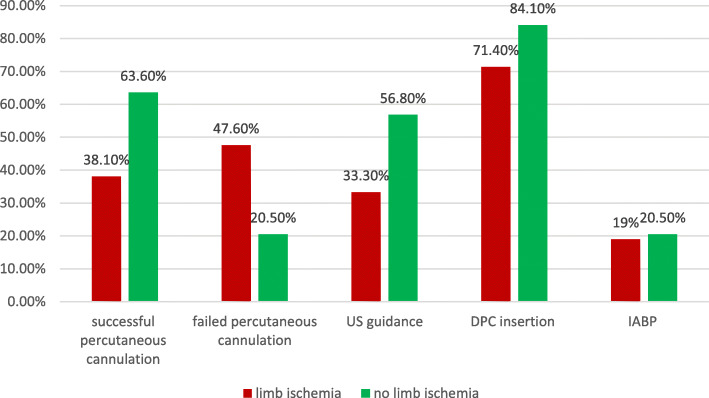
Cannulation approaches of femoral VA-ECMO support

**Fig. 3:**
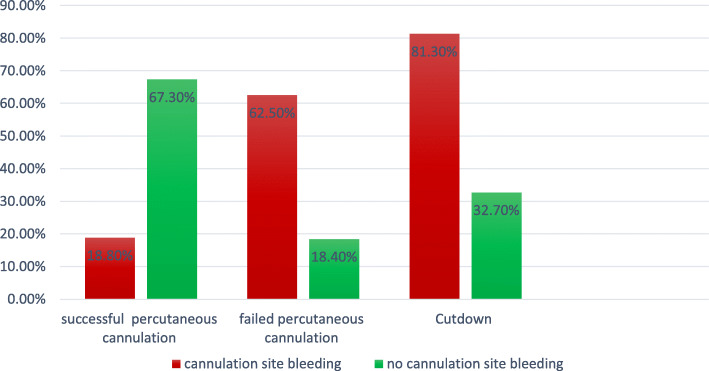
Cannulation site bleeding of the studied VA-ECMO-treated patients

The occurrence of acute limb ischemia was significantly correlated with failed percutaneous femoral cannulation (*p* = 0.039) as failed insertion occurred in 47.6% vs 20.5%, while successful insertion occurred in 38.1% vs 63.6% in the patients with and without limb ischemia, respectively. There was no significant statistical correlation between the cut-down technique and occurrence of limb ischemia (*p* = 0.053). The occurrence of femoral cannulation site bleeding was significantly correlated with failed percutaneous cannulation (*p* = 0.001) and cut-down technique (*p* = 0.001). Failed percutaneous insertion occurred in 62.5% vs 18.4%, while the cut-down technique was done in 81.3% vs 32.7% of the patients with and without cannulation site bleeding, respectively (Tables 1 and 5, Figs. 2 and 3).

**Table 5:** The correlations between cannulation site bleeding and cannulation techniques

Variables	Cannulation site bleeding	No site bleeding	*P* value
Percutaneous cannulation			
Successful	3 (18.8%)	33 (67.3%)	0.001
Failed	10 (62.5%)	9 (18.4%)	
Not tried	3 (18.8%)	7 (14.3%)	
Cut-down			
Done	13 (81.3%)	16 (32.7%)	0.001
Not done	3 (18.8%)	33 (67.3%)

## Discussion

We retrospectively analysed our adult patients who had femoral VA-ECMO support for refractory cardiogenic shock including post-cardiotomy shock in 26.2% of patients. We observed acute lower limb ischemia in 32.3% of cases and cannulation site bleeding in 24.6% of cases while bleeding required vascular exploration happened in 6.3% of cases. We reviewed the recent literature about the vascular complications after VA-ECMO and found a wide range of incidence which may be related to different patients’ demographic data, indications and cannulation techniques of VA-ECMO and use of distal perfusion cannulae [8, 10, 17–20].

Our incidence of acute limb ischemia was similar to Yen et al.’s study that reported acute limb ischemia in 33% of VA-ECMO-treated patients, even with the use of DPC [8]. Our results were different from Yang et al. [18] study that reported only 8.6% incidence of acute lower limb ischemia. That difference may be related to the difference of studied patients as that study included only patients with post-cardiotomy shock and the ECMO cannulae were inserted by cut-down approach only with concomitant prophylactic DPC insertion. Tanaka et al. reported 20% incidence of acute vascular complications included a 12% incidence of acute limb ischemia with compartmental syndrome requiring fasciotomy, even in the presence of DPC [10]. Gander et al.’s study reported 52% incidence of limb ischemia requiring surgical interventions and 81% of those patients had prophylactic DPC [21].

The development of acute limb ischemia can occur at ECMO initiation, support, or after decannulation due to multifactorial reduced blood flow to the limb especially in absence of collateral circulation. The use of relatively large-sized arterial cannulae in relation to the arterial femoral artery diameter or BSA was associated with acute limb ischemia in some studies [6, 22]. Few studies reported that younger patients have higher risk of acute limb ischemia due to smaller femoral arteries and lack of collateral circulation compared to the elderly [23]. Also, female patients have higher risks compared to men due to smaller arterial sizes [10, 24]. In our study, we did not find any significant  difference between both groups regarding patient age, sex, weight, height, BMI, nor BSA. Yang et al. study reported absence of age or sex differences but the patients who developed vascular complications were significantly obese [18].

Our results revealed that percutaneous approach was tried in 84.6% of patients but was successful in only 55.4% of patients. The occurrence of acute limb ischemia was significantly correlated with failed percutaneous femoral cannulation. The occurrence of femoral cannulation site bleeding was significantly correlated with failed percutaneous cannulation and cut-down technique. The percutaneous approach was the most used technique in many studies evaluating femoral VA-ECMO [8, 10, 25–28]. The percutaneous cannulation is characterized by being rapid easy approach with reduced risk of cannulation site bleeding but its main disadvantages include possibilities of vascular injuries and failed intraluminal catheter placement. Moreover emergent percutaneous cannulation in patients with haemodynamics collapse is a major challenge that may be complicated with vascular complications [29, 30]. The use of ultrasound guidance in getting femoral access with first pass success was reported in many studies comparing it with the landmark techniques [31–33]. Kashiura et al. [34] recommended a combination of ultrasound guidance to get femoral access then fluoroscopy to ensure correct intraluminal guiding wire placement before dilators use and cannulae insertion. That study reported fewer vascular complications with this combination than ultrasound guidance alone without a significant delay of ECMO flow initiation.

The cut-down technique is usually done after cardiotomy with failed weaning off cardiopulmonary bypass. In our study, the cut-down technique was done mainly after failed percutaneous approach, so we could not compare between the 2 approaches regarding the vascular complications and outcomes. Slottosch et al. [35] reported the fewer vascular complications associated with the surgical approach compared with the percutaneous cannulation. However, Danial et al. [36] reported absence of significant difference between both approaches regarding acute limb ischemia in the same centre but the percutaneous approach was associated with significant bleeding requiring surgical intervention after decannulation.

Eighty percent of our studied patients had prophylactic DPC insertion with ECMO initiation and there was no significant difference between both groups. The prophylactic DPC insertion was done as a preventive strategy to avoid significant limb ischemia with variable results in many studies [8, 10, 18, 21, 37, 38]. Few trials were done to identify the patients who need a DPC instead of prophylactic insertion. Huang et al. [39] reported successful use of DPC if invasive mean arterial blood pressure was less than 50 mmHg in femoral artery distal to the arterial cannula of ECMO. Near-infrared spectroscopy (NIRS) was used to identify the regional limb tissue oxygenation (rSO_2_%) and help to detect ischemia even with non-pulsatile blood flow. Schachner et al. [40] used NIRS monitoring and reported  a drop of tissue oxygenation from 61 to 38% and going back to normal baseline values after DPC insertion. Wong et al. [41] used NIRS to concomitantly monitor brain and limb regional oxygenation. They reported clinically significant vascular events when rSO_2_% decreased below 40% or more than 25% decrease from baseline values.

According to our results and compared to patients without acute limb ischemia, the patients who developed acute ischemia had significant AKI and longer ICU stay but without significant haemodialysis, cerebral strokes, nor hospital mortality. Yang et al. [18] reported similar cerebrovascular stokes and renal replacement therapy but fewer ICU stay and fewer hospital mortality in the patients who developed acute vascular complications compared to the patients without vascular complications after peripheral VA-ECMO for post-cardiotomy shock via femoral cut-down approach. Gander et al. [21] reported absence of mortality difference between the patients with and without acute limb ischemia. Finally according to our study, we think that the emergent femoral percutaneous cannulation in patients with haemodynamics instability and coagulopathy could result in failed cannulation trials which were associated with the acute limb ischemia and cannulation site bleeding. We could recommend a careful approach and using image modalities during the whole process of femoral cannulation, guidewire introduction and cannulae insertion, and then close monitoring of limb perfusion.

## Conclusion

Acute vascular complications are frequent after femoral VA-ECMO. Failed percutaneous femoral cannulation has been, in this study identified as the most important risk factor for acute limb ischemia and cannulation site bleeding. A careful approach during femoral cannulation is recommended to prevent occurrence of acute limb ischemia and femoral cannulation site bleeding.

### Limitations

Our work was a single-centre retrospective study with a relatively limited number of patients.

## Data Availability

The data used in this study are available from the corresponding author upon a reasonable request.

## Abbreviations

AKI: Acute kidney injury
AF: Atrial fibrillation
aPTT: Activated partial thromboplastin time
BSA: Body surface area
BMI: Body mass index
CKD: Chronic kidney disease
CI: Cardiac index
CVS: Cerebrovascular stroke
DPC: Distal perfusion cannula
EF: Ejection fraction
LVAD: Left ventricle assist device
MCS: Mechanical circulatory support
NIRS: Near-infrared spectroscopy
IABP: Intra-aortic balloon pump
INR: International normalized ratio
SOFA: Sequential organ failure assessment
US: Ultrasound
VA-ECMO: Veno-arterial extracorporeal membrane oxygenation
